# Investigating the Mechanisms of Jieduquyuziyin Prescription Improves Lupus Nephritis and Fibrosis via FXR in MRL/lpr Mice

**DOI:** 10.1155/2022/4301033

**Published:** 2022-07-09

**Authors:** Jingqun Liu, Qing Ma, Qice Sun, Qihan Luo, Yiheng Wang, Cheng Wang, Akao Zhu, Lisha Zhao, Lu Yin, Jiang Lou, Yu Dong, Ping Qiu

**Affiliations:** ^1^The First College of Clinical Medicine, Zhejiang Chinese Medical University, Hangzhou, China; ^2^The Second School of Zhejiang Chinese Medical University, 310053, China; ^3^School of Pharmacy, Zhejiang Chinese Medical University, Hangzhou, China; ^4^Academy of Chinese Medical Science, Zhejiang Chinese Medical University, Hangzhou, China; ^5^The Second Affiliated Hospital of Zhejiang University School of Medicine, Hangzhou, Zhejiang, China; ^6^Department of Medicine, Zhejiang Academy of Traditional Chinese Medicine, Hangzhou 310007, China; ^7^Department of Pathology, Affiliated Hangzhou First People's Hospital, School of Medicine, Zhejiang University, Hangzhou, China; ^8^Department of Pharmacy, Affiliated Hangzhou First People's Hospital, Zhejiang University School of Medicine, Hangzhou, Zhejiang, China

## Abstract

Lupus nephritis (LN) is one of the most serious complications of systemic lupus erythematosus (SLE) and one of the leading causes of death. An alternative effective treatment to ameliorate and relieve LN and delay the process of renal tissue fibrosis is urgently needed in the clinical setting. Jieduquyuziyin prescription (JP) has been successfully used to treat SLE, but its potential mechanisms are not sufficiently understood. In this study, we treated MRL/lpr mice with JP for 8 weeks and treated human renal tubular epithelial cells (human kidney 2 (HK-2)) with drug-containing serum to observe the antagonistic effects of JP on inflammation and fibrosis, as well as to investigate the possible mechanisms. Results demonstrated that JP significantly reduced urinary protein and significantly improved pathological abnormalities. Metabolomics combined with ingenuity pathway analysis illustrated that the process of kidney injury in lupus mice may be closely related to farnesoid X receptor (FXR) pathway abnormalities. Microarray biomimetic analysis and LN patients indicated that FXR may play a protective role as an effective therapeutic target for LN and renal fibrosis. JP significantly increased the expression of FXR and inhibited the expression of its downstream targets, namely, nuclear transcription factor *κ*B (NF-*κ*B) and *α*-smooth muscle actin (*α*-SMA), in the kidney of MRL/lpr mice and HK-2 cells, as confirmed by in vitro and in vivo experiments. In conclusion, JP may mediate the activation of renal FXR expression and inhibit NF-*κ*B and *α*-SMA expression to exert anti-inflammatory and antifibrotic effects for LN prevention and treatment.

## 1. Introduction

Systemic lupus erythematosus (SLE) has a prevalence of 12–39/100000 on the global average and close to 1/1000 among women [1]. SLE is an autoimmune disease in which pathogenic autoantibodies and immune complexes form and inflict damage to organs and tissues, often with multisystem involvement. According to clinical statistics, lupus nephritis (LN), one of the most serious complications of SLE, can progress to renal failure in advanced stages and is an important cause of death. LN pathogenesis is complex and may be closely related to immune dysfunction, leading to the formation of autoantibodies that activate the complement system by circulating immune complexes, as well as the extensive involvement of inflammatory cells and cytokines. Currently, the clinical treatment of LN is based mostly on therapies such as immunosuppressants or hormones suppress the immune responses to “self-antigen,” which cannot completely alleviate the disease progression [2]. Moreover, long-term and high-dose use can cause many toxic side effects [3], leading to a serious impact on the survival and quality of life of patients. Therefore, efforts to identify an alternative therapy that effectively improves LN with few adverse effects are in urgent clinical demand.

Traditional Chinese medicine (TCM) for SLE treatment is receiving considerable attention for its high efficacy and ability to attenuate toxicity. In the present study, Jieduquyuziyin prescription (JP) was derived from “Sheng Ma Bie Jia Tang,” a frequently used Chinese formula described by Zhang Zhongjing in the synopsis of prescriptions of the golden chamber. In clinical practice, this formula is used to treat patients with hyperactivity of fire due to Yin deficiency syndrome and lupus patients with abnormal renal function. Several studies have shown that this formula has been successfully applied clinically to treat SLE in preclinical models or clinical settings [4]. Notably, a multicenter, large-sample randomized controlled clinical trial has confirmed the clinical benefits of this formula in improving the condition of SLE patients and reducing their hormonal toxicities [5]. Previous studies have indicated that JP could slow down the progression of lupus disease by ameliorating immune function in lupus model mice [6, 7]. For instance, the JP formula significantly reduces urinary proteins, serum dsDNA, and creatinine level, as well as significantly decreases periglomerular inflammatory cell infiltration and the thickening of the glomerular basement membrane in MRL/lpr mice. These results suggest that JP may exert anti-inflammatory and antifibrotic effects to improve LN but the underlying mechanisms require further exploration.

To elucidate the mechanism behind the improvement of LN by JP, we used a combined analysis of serum metabolomics and ingenuity pathway analysis (IPA) database to evaluate the pharmacological effects of JP and its potential target pathways. In recent years, metabolomics has been extensively used in the field of TCM and the efficacy of TCM herbal medicines can be explained in the scientific language of metabolomics and serum medicinal chemistry [8, 9]. IPA is a cloud-based graphical interface bioinformatics software that can analyze, integrate, and understand histological data from the perspective of biological pathways. It is suitable for large-data analysis such as transcriptomics, proteomics, and metabolomics, as well as for some small-scale ones that generate lists of genes and chemical-substance experiments. IPA combined with metabolomics can predict targets and related pathways [10, 11]. Accordingly, this study conducted integrated research at overall, cellular, and molecular levels to reveal the anti-inflammatory and antirenal fibrotic mechanisms of JP. The aim was at providing new targets and strategies for anti-LN drug research and theoretical basis for SLE clinical treatment.

## 2. Materials and Methods

### 2.1. Drugs and Reagents

The composition of original JP herbal medicine was determined according to the weight ratio of 5 : 5 : 5 : 4 : 4 : 4 : 3 : 3 : 2 by combining *Rehmanniae radix*, *Herba hedyotidis diffusae*, Paeoniaeradix rubra, *Trionycis carapax*, *Artemisiae annuae herba*, *Centellae herba*, *Semen Coicis*, *Citri sarcodactylis fructus*, *Cimicifugae rhizoma*, and *Glycyrrhizae radix* et rhizome. Ten drugs were beaten, treated, soaked for 1 h, and boiled for 1 h. The filtrate was collected, and the residue was boiled again for 1 h. The combined filtrates were concentrated to 2 g/mL raw drug.

### 2.2. UHPLC-Q/TOF-MS System Analysis of JP

The instruments used were a SCIEX X-500R quadrupole time-of-flight mass spectrometer (AB SCIEX, USA), a TurboIonSpray ion source (AB SCIEX, USA), and a Waters ACQUITY I-Class Plus UPLC ultraperformance liquid chromatography system (Waters, USA). The chromatographic conditions were as follows: ACQUITY UPLC BEH C18 (150 × 2.1 mm^2^, 1.7 *μ*m) column, mobile phase, and 0.1% formic acid acetonitrile (A)–0.1% formic acid water (B). The gradient-elution program was as follows: 0~4 min, 99%–87% B; 4–14 min, 87%–80% B; 14–20 min, 80%–50% B; 20–24 min, 50–10% B–24 min, 50%–10% B; 24–24.5 min, 10%–1% B; 24.5–28 min, and 1% B. The flow rate was 0.3 mL/min, the injection-tray temperature was 8°C, the column temperature was 40°C, and the injection volume was 2 *μ*L. Time-of-flight mass spectrometry was performed with a TurboIonSpray ion source and the ESI positive- and negative-ion scanning mode.

SCIEX OS software was used to acquire and process the data. The screening of the target compounds can be completed without standards by searching the secondary database of the TCM MS/MS Library (containing more than 1000 secondary data of TCM compounds) configured by SCIEX OS based on the exact mass number of the compounds at the primary level, isotope-distribution ratio, and MS/MS.

### 2.3. Animals and Experimental Design

SPF-grade MRL/lpr and MRL/MPJ mice purchased from the animal experiment center of Zhejiang University of Traditional Chinese Medicine were housed in a barrier environment and given a standard feeding environment and diet. Each group comprised six mice. MRL/MPJ mice (*n* = 6) treated with saline were used as controls. Twelve MRL/lpr mice were randomly divided into two groups, namely, saline-treated and JP groups (36 g/kg body weight per day, i.g.).The gavage doses of JP in the animal experiment were according to the clinical dose, which was converted into an equivalent dosage.

Blood was collected from the eye and 8 weeks at 3000 rpm/min, and serum was collected and dispensed. After decapitation, the tissue samples were frozen and one side of the kidney was fixed. The samples were frozen at −80°C in the refrigerator for subsequent analysis.

### 2.4. Determination of Biomarkers

Urine was collected for 24 h using a metabolic cage the day before the mice were executed. Urine protein was measured using a Bradford Protein Assay Kit (Catalog No. P0006; Beyotime, China). Anti-nuclear antibody (ANA) and anti-double-stranded DNA (dsDNA) antibody were measured using a Mouse ANA ELISA Kit (catalog no. F9253-A; FANKEWEI, China) and Mouse Anti-dsDNA Antibody ELISA Kit (catalog no. F30389-A; FANKEWEI, China).

### 2.5. Histopathological Analysis of Kidney Tissue

Some kidney tissues fixed in formalin were dehydrated in alcohol gradient and then immersed in wax. The wax-immersed tissues were embedded, frozen, and trimmed, and then, the cooled wax blocks were placed in a paraffin-sectioning machine. Sections 4 *μ*m thick were fixed on slides. Afterwards, the sections were stained with hematoxylin eosin, Masson stain, or PASM stain. Finally, sections were scanned and observed for renal histopathology.

### 2.6. IgG Immunofluorescence Staining

Dewaxing and in-hydration processes were performed using xylene and gradient alcohol. Antigen repair was performed using EDTA antigen repair buffer. After BSA closure for 30 min, the primary antibody was incubated overnight at 4°C, followed by three PBS washes for 5 min. The secondary antibody of the corresponding species was used to incubate at room temperature for 1 h. Cell nuclei were restained with DAPI and blocked with antifluorescence quencher and scanned or photographed for observation.

### 2.7. Immunofluorescence Analysis of Farnesoid X Receptor (FXR) and *α*-Smooth Muscle Actin (*α*-SMA) in Kidney Tissues

Paraffin sections were dewaxed and hydrated for antigen repair and fixed in circles, closed with hydrogen peroxide for 25 min, and closed with BSA for 30 min. Primary antibodies were incubated overnight at 4°C and washed three times. The corresponding HRP-labeled secondary antibody was incubated for 1 h at room temperature, and cy3-TSA (or FITC-TSA) was added and incubated for 10 min in darkness. Microwave treatment was used to remove the bound primary and secondary antibodies. The second protein was labeled by the same method. Finally, Hoechst-stained nuclei were sealed and fluorescence scan or microscopic photo-observation was performed.

### 2.8. Serum Metabolomics

We thawed 200 *μ*L of the serum sample on ice at room temperature and added 600 *μ*L of methanol/ethanediol (1 : 1) prechilled at −20°C overnight. After vortexing, the supernatant was placed in a centrifuge tube and dried in a vacuum centrifuge concentrator. After drying the sample, acetonitrile/methanol (80/20)–water mixture (1 : 1) was added, and then, the mixture was redissolved, vortexed for 60 s, and centrifuged at 14000 r/min for 15 min at 4°C. We collected 4 *μ*L of supernatant for UPLC-Q/TOF-MS analysis. The raw data in .swiff2 were converted to .mzXML format by using ProteoWizard. The XCMS offline workstation was used for peak alignment, retention-time correction, and extraction of peak areas. Multidimensional statistical analysis was performed using SIMCA-P 13.0 (Umetrics, Umea, Sweden), including unsupervised principal component analysis, supervised partial least square discriminant analysis (PLS-DA), and orthogonal PLS-DA.

### 2.9. Kidney Transcriptomics

RNA from total samples was isolated and purified using TRIzol (Invitrogen, CA, USA). Total RNA was then quality controlled using NanoDrop ND-1000 (NanoDrop, Wilmington, DE, USA). RNA integrity was examined with a Bioanalyzer 2100 (Agilent, CA, USA) and verified by agarose electrophoresis. Concentrations > 50 ng/*μ*L, RIN values > 7.0, OD260/280 > 1.8, and total RNA > 1 *μ*g satisfied the downstream experiments. A library with a fragment size of 300 bp ± 50 bp was formed by PCR–predenaturation held at 95°C for 3 min, denaturation at 98°C for a total of 8 cycles of 15 s each, annealing to 60°C held for 15 s, extension at 72°C for 30 s, and final extension held at 72°C for 5 min. Finally, the library was double-ended sequenced using Illumina NovaSeq™ 6000 (LC Bio Technology Co. Ltd., Hangzhou, China) in the PE150 sequencing mode according to the standard operation. Data were cleaned by removing low-quality sequences and duplicates to obtain CleanData in fastq.gz format. The Ballgown package was used to provide file input for FPKM quantification. Significant differences between samples were determined using the R package edgeR.

### 2.10. IPA and GEO Screen JP to Improve LN Pathways and Key Targets

IPA is an online analysis software package based on scientific data collected by the Ingenuity Knowledge Base. It can help elucidate drugs and the network of their interactions. The differential biomarkers obtained in this experiment after JP intervention and their properties and corresponding variables were analyzed with IPA software, which can effectively visualize the potential targets of JP intervention in SLE.

To identify the major DEGs between normal human kidney and LN specimens, microarray data (GSE32592, GSE60861, GSE99340, and GSE104948) were obtained from the Gene Expression Omnibus (http://www.ncbi.nlm.nih.gov/geo/) along with appropriate microarray annotation data. DEG was defined by the limma package in the R statistical environment. *P* < 0.01 and a threshold value of ≥1.5 for fold change |FC| were applied. Known targets associated with LN were obtained from five currently available databases using “lupus nephritis” as a keyword: (1) DrugBank (http://www.drugbank.ca/), (2) OMIM (http://www.omim.org/), (3) GAD (http://geneticassociationdb.nih.gov/), (4) TTD (http://database.idrb.cqu.edu.cn/TTD/), and (5) PharmGKB (https://www.pharmgkb.org/index.jsp).

### 2.11. Total RNA Extraction and qPCR Analysis

Total RNA was extracted from mouse kidney tissues using TRIzol reagent (Invitrogen, Carlsbad, CA, USA). EzOmicsTM One-Step qPCR Kit (Bioomics, China) and Lightcycler 96 (Roche, Basel, Switzerland) were used for mouse samples. The primer sequences were as follows: *Nr1h4*, 5′-CATCAAGGACAGAGAGGCGG-3′ and 5′-TCAGCGTGGTGATGGTTGAA-3′; *Acta2*, 5′-GCTACGAACTGCCTGACGG-3′ and 5′-GCTGTTATAGGTGGTTTCGTGGA-3′; and *Gapdh*, 5′-GTGTTCCTACCCCCAATGTGTGT-3′ and 5′-ATTGTCATACCAGGAAATGAGCTT-3′. The relative expression levels of the target mRNAs were calculated by the 2(−∆∆*CT*) method and then normalized. The experiments were performed in triplicate.

### 2.12. Western Blot (WB) Analysis

Frozen kidneys were homogenized and extracted in RIPA lysis buffer (Beyotime, P0013C, Shanghai, China) and then boiled and centrifuged for 10 min to obtain the supernatant. Harvested HK-2 cells were processed in the same manner. Samples containing equal amounts of protein were separated on 10% SDS-PAGE and transferred to PVDF membranes. The membranes were incubated overnight at 4°C with one of the following primary antibodies: primary antibodies including anti-FXR (santa, sc-25309; 1 : 1000), anti-p65 (affinity, AF5006; 1 : 1000), anti-GAPDH (affinity, AF7021; 1 : 3000), anti-I*κ*B (Beyotime, AF1282; 1 : 1000), anti-laminin B1 (affinity, AF5161; 1 : 1000), anti-tubulin *β* (affinity, AF7011; 1 : 1000), anti-*α*-SMA (affinity, AF1032; 1 : 1000), and anti-p-I*κ*B (Beyotime, AF5839; 1 : 1000). After washing three times, the membranes were washed with peroxidase-AffiniPure Goat Anti-Rabbit IgG (cat# 111-035-003, Jackson ImmunoResearch) and peroxidase-AffiniPure Goat Anti-Mouse IgG (cat# 115-035-003, Jackson ImmunoResearch) diluted at 1 : 1000–1 : 3000. After incubation at room temperature for 2 h, the membranes were washed three times. The experiments were performed in triplicate. Images were captured using the provided Odyssey software v3.0. Blots were quantified using ImageJ software (ImageJ 1.34 s). Quantization results are represented by the gray value and normalized to GAPDH, Lamin B1, or tubulin *β*. All the complete Western blot band images are provided in the supplementary materials (available here).

### 2.13. ELISA

A human TNF-*α* ELISA Kit (ABclonal, cat no: RK0030) and a human IL-6 ELISA Kit (ABclonal, cat no: RK00004) were used to detect the TNF-*α* IL-6 cytokine levels in a medium with HK-2 cells transfected and treated with drug-containing serum.

### 2.14. Preparation of JP Serum

Clean SD rats each weighing 200 g purchased from the animal experiment center of Zhejiang University of Traditional Chinese Medicine were used. The rats were randomly divided into control and treatment groups by gavage of distilled water and JP, respectively. The dose was determined according to the conversion between clinical human and rat doses of 125 g/kg. After 5 days of treatment, the rats were anesthetized and blood was collected from the abdominal aorta. The blood was centrifuged at 3000 rpm/min for 20 min at room temperature. Afterwards, the serum was extinguished at 56°C for 30 min, passed through a 0.22 *μ*m filter head, and stored in a −80°C refrigerator.

### 2.15. Cell Culture

Human immortalized HK-2 cells were purchased from Procell Life Science & Technology Co. The cells were cultured in PRMI 1640 medium (Gibco, ref: C11875500BT) at 5% CO_2_ and 37°C supplemented with 10% fetal bovine serum (Multicell, cat no: 086-150) and 1% penicillin/streptomycin (Biosharp, cat no: BL505A). When HK-2 cells reached 80%–90% confluence, they were collected with trypsin solution (Biosharp, cat no: BL512A) and inoculated in 24-well plates at a density of 8 × 10^4^ cells per well. The plates were incubated for 24 h before other treatments.

### 2.16. Transfections of Plasmids and Small Interfering RNA (siRNA)

Plasmid *NR*1*H*4 − 3∗Flag (cat# 26786GZ-P) was obtained from Genomeditech (Shanghai, China). siRNA for *NR1H4* was purchased from GenePharma (GenePharma, Shanghai, China). The following were the specific sequences of *NR1H4*-siRNA: justification: GUACUCUCCUGGAAUAUAUTT and antisense: AUAUAUUCCAGGAGGAGUACTT. HK-2 was cultured in 24-well plates for 24 h prior to transfection, and then, plasmids and siRNAs were transfected into cells according to the jetPRIME Kit (Polyplus-transfection SA, Illkirch-Graffenstaden, France) protocol. TGF-*β*1 (Abbkine, cat#: PRP100190—5 *μ*g) was reconstituted with provided cytokine buffer, and the storage concentration was 100 *μ*g/mL. About 24 h after transfection, it was stimulated with 20 ng/mL TGF-*β*1 for 24 h and the cells were digested with trypsin and collected. HK-2 cells in 24-well plates were transfected with FXR overexpression plasmid or siRNA. After 24 h of transfection, the medium was changed and the cells were cultured for 48 h and stimulated with 1 *μ*g/mL LPS (Sigma, L2880—10 mg) for 6 h. The cell supernatant was collected to detect the content of cytokines IL-6 and TNF-*α*. After transfection for 24 h, HK-2 cells were stimulated with TGF-*β* 20 ng/mL for 24 h to induce renal tubular fibrosis and then grouped them into FXR overexpression, FXR siRNA transfection, control siRNA, and 10% concentration JP-medicated serum. Subsequently, the cells were collected to detect FXR and *α*-SMA protein levels by WB. After the cells were transfected with or FXR/*α*-SMA siRNA for 24 h and treated with TGF-*β* for 24 h, they were collected to detect E-cadherin and vimentin protein levels to evaluate the occurrence of the epithelial-to-mesenchymal transition (EMT) process.

### 2.17. Statistical Analysis

All data are expressed as the mean ± SD and analyzed using GraphPad Prism (version 8.0.1) software. All statistical comparisons were performed using one-way ANOVA, and Dunnett's test was used to correct for multiple comparisons. Values of *P* < 0.05 were considered statistically significant.

## 3. Results

### 3.1. JP Composition and UPLC-Q/TOF-MS Analysis

**Figure 1:**
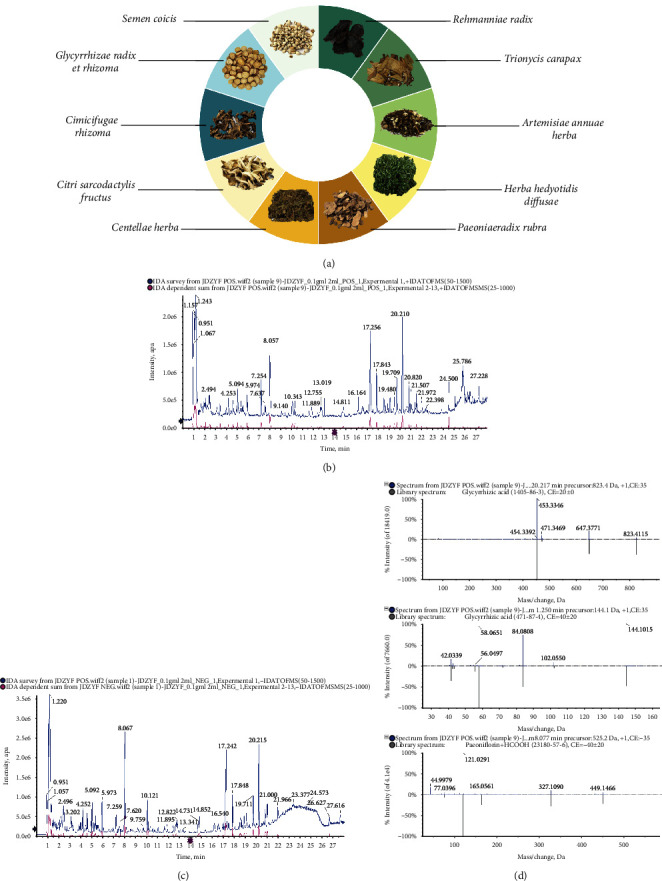
Composition of the traditional Chinese medicine JP. (a) Total ion flow diagram of UPLC-Q/TOF-MS (b) positive-ion and (c) negative-ion modes. (d) Identification of some components.

The JP composition of 10 Chinese herbal medicines is shown in Figure 1(a). The water extract of JP was detected with the UPLC-Q/TOF-MS instrument. The total-ion flow chart is shown in Figures 1(b) and 1(c). By comparing and screening the first-order accurate mass number, isotope-distribution ratio, and MS/MS in the secondary database of TCM MS/MS provided by SCIEX OS software, we found that the prescription primarily comprised glycyrrhizic acid, glycyrrhizin, paeoniflorin, iso-chlorogenic acid, chlorogenic acid, oleoside, hydroxyasiaticoside, deacetylcyrrhizic acid, asiaticoside, isoferulic acid, and so on. The identification process of some compounds is shown in Figure 1(d).

### 3.2. JP Reduced Serum Autoantibody Levels and Mitigated Kidney Damage in MRL/lpr Mice

**Figure 2:**
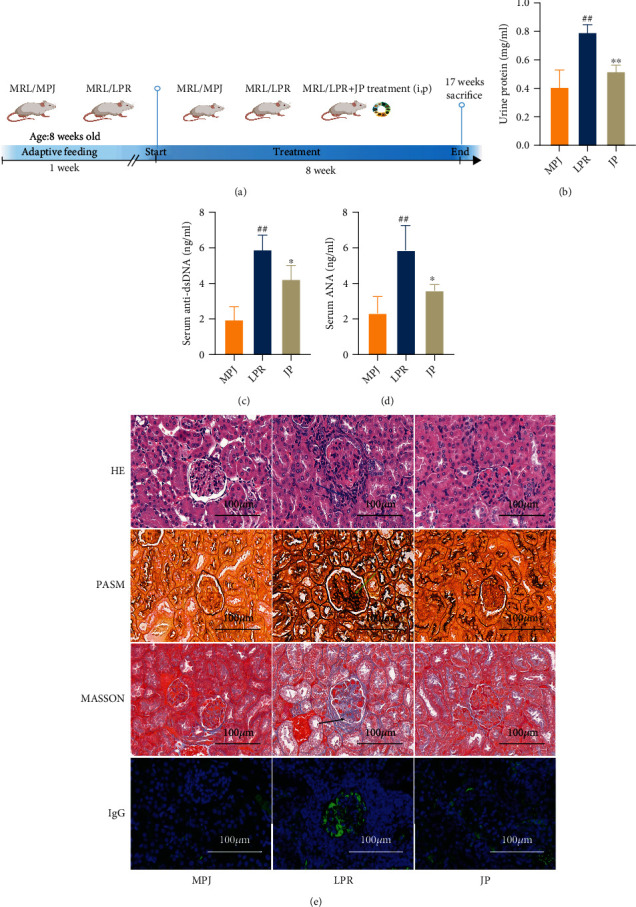
Urinary protein, serum autoantibody level, and renal pathology. The indices include urinary protein (a), anti-dsDNA antibody (b), and ANA (c). Renal histopathology (d) included periglomerular inflammatory cell infiltration (HE), glomerular mesangial hyperplasia (PASM), mesangial fibrosis (Masson), and glomerular IgG deposition (scale, 100 *μ*m). The control group was MRL/MPJ mice and the model group was MRL/lpr mice. Data are expressed as the mean ± standard deviation (*n* = 6). ^#^*P* < 0.05 and 0.01 versus the MPJ group; ^∗#^*P* < 0.05, *P* < 0.05, and *P* < 0.01 versus the lpr group.

Results demonstrated that JP reduced the levels of urinary protein (*P* < 0.01; Figure 2(a)), anti-dsDNA antibody (*P* < 0.01; Figure 2(b)), and ANA (*P* < 0.01; Figure 2(c)) in the serum of mice. Histopathological staining of mouse kidneys (Figure 2(d)) suggested that JP reduced periglomerular inflammatory cell infiltration, as well as glomerular thylakoid proliferation and fibrosis. It also decreased IgG immune-complex deposition in lupus-like mice. Overall, our results indicated that auto-antibodies in the serum of JP-treated mice significantly decreased and the inflammation and fibrosis of renal tissue improved.

### 3.3. Serum Metabolic Changes after Treatment with JP

**Figure 3:**
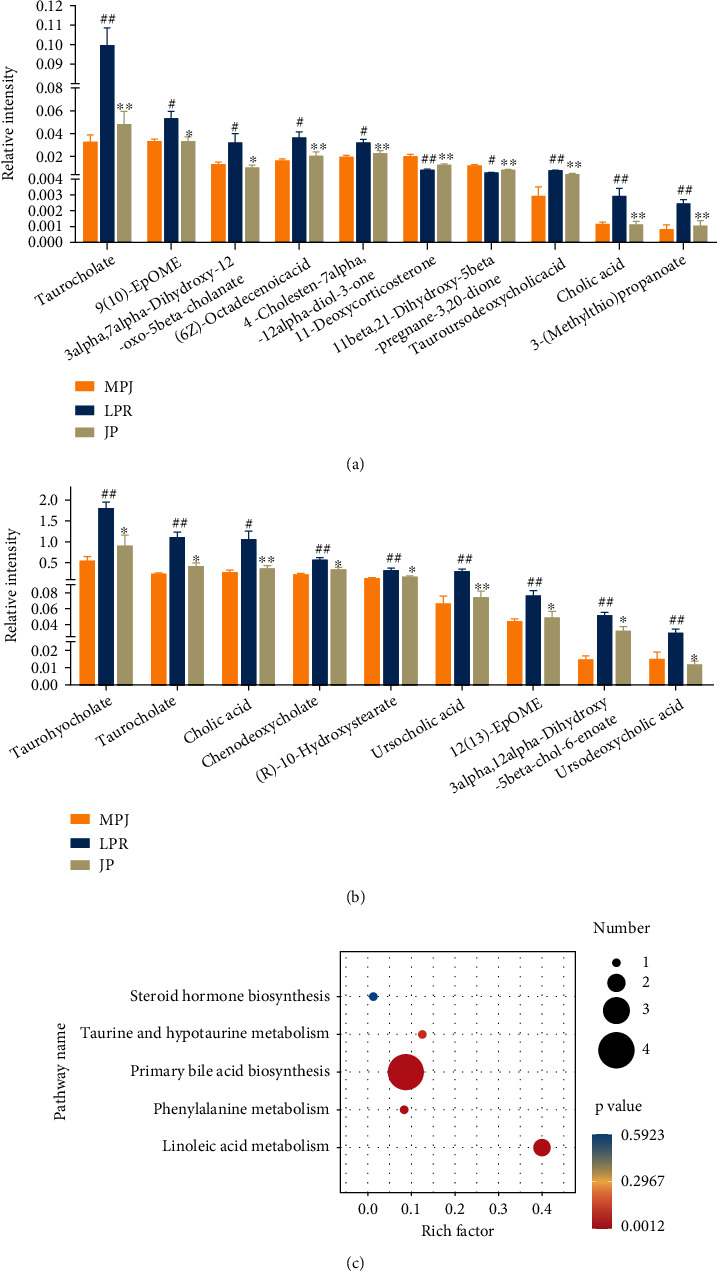
Serum metabolomics of mice with JP backregulated metabolites and metabolic pathway enrichment analysis. The differential metabolites of mouse serum were analyzed by chromatography–mass spectrometry, and the differential metabolites were screened in positive-ion (a) and negative-ion (b) modes and enriched for metabolic pathways (c). Data are expressed as the mean ± standard deviation (*n* = 6). ^#^*P* < 0.05 and ^##^*P* < 0.01 versus the MPJ group; ^∗^*P* < 0.05 and ^∗∗^*P* < 0.01 versus the lpr group.

To further elucidate the mechanism of JP, we subjected the serum of each group of mice to a nontargeted metabolomic study and screened the differential metabolites that could be significantly backregulated by JP in positive- or negative-ion modes (Figures 3(a) and 3(b)). Metabolic pathway analysis (Figure 3(c)) showed that JP treatment could induce changes in the metabolic pathways of primary bile acid biosynthesis, linoleic acid metabolism, steroid hormone synthesis, and phenylalanine metabolism in MRL/lpr mice.

### 3.4. JP Modulated FXR-Related Pathways to Improve LN

**Figure 4:**
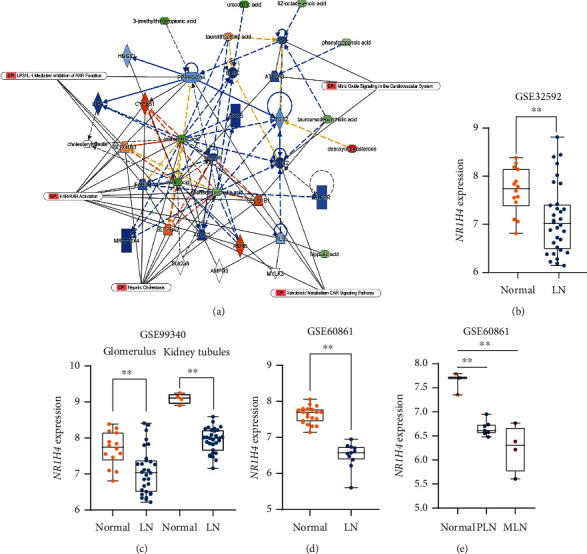
Ingenuity pathway analysis and GEO database combined to analyze serum differential metabolite-related pathways and targets. IPA software was used to analyze the core pathways affected by differential metabolites between JP and lpr groups (a). GEO database was used to determine the differential expression of pathway-related proteins in lupus patients (b). Data are expressed as the mean ± standard deviation (*n* ≥ 3), ^∗∗^*P* < 0.01.

To further explore the target of JP antagonism of LN, we predicted the target pathway analysis through serum metabolomics combined with IPA database. Results suggested that the mechanism of action may be closely related to the FXR (Figure 4(a)). We further confirmed through the results of GEO data analysis that FXR (*NR1H4*) expression obviously decreased in the kidneys of patients with LN (*P* < 0.01; Figures 4(b)–4(d)) and the degree of inhibition was strongly associated with disease progression and poor prognosis (*P* < 0.01; Figure 4(e)).

### 3.5. Nuclear Factor *κ*B (NF-*κ*B) and *α*-SMA as Possible FXR Downstream Targets

**Figure 5:**
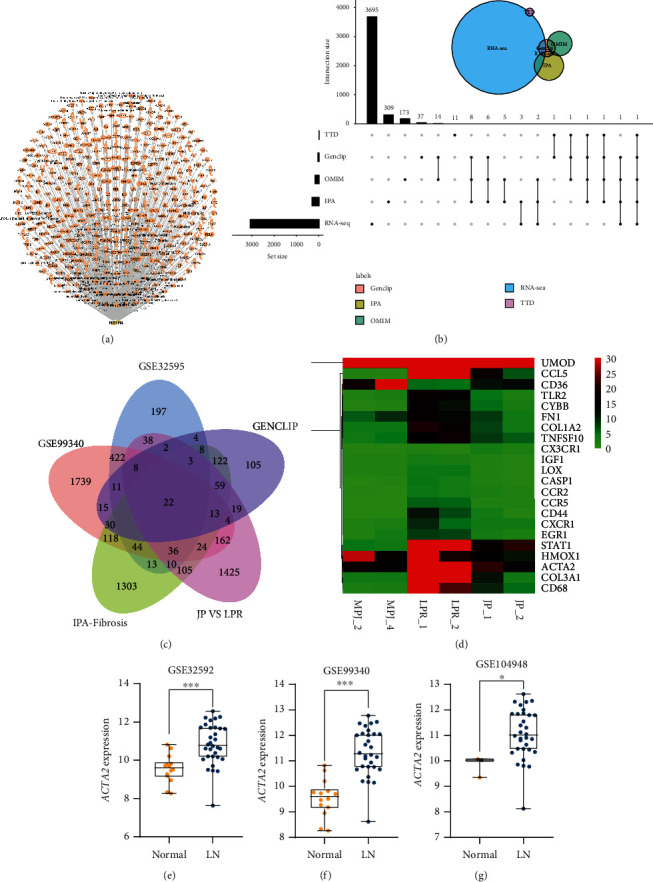
Prediction of downstream inflammatory and fibrotic targets of FXR in LN. Combination of the downstream targets of FXR predicted using IPA software (a) with the disease target database of LN and differential genes from transcriptome sequencing to screen for possible downstream inflammatory targets of FXR by upset Venn diagram (b). Wenn plots (c) for screening FXR downstream fibrosis-related targets from GEO database, disease target database, and transcriptome-sequencing data for disease–target intersection analysis; they are presented by the heat map of transcriptome data (d). Box plots (e) showing the differential expression of the focused targets in GEO database. Data are expressed as the mean ± standard deviation (*n* ≥ 3), ^∗^*P* < 0.05, ^∗∗^*P* < 0.01.

**Figure 6:**
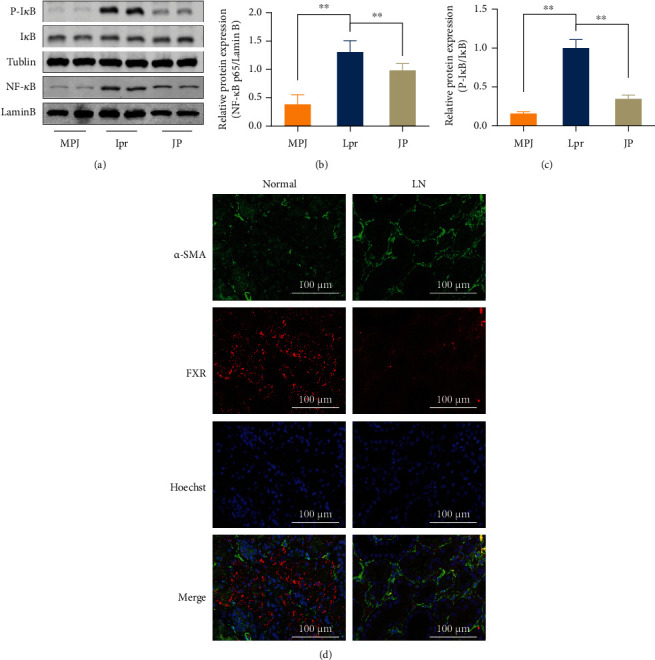
NF-*κ*B nuclear transfer decreased in the mouse kidney. The relationship between FXR and *α*-SMA regulation was investigated through in vitro experiments. WB results (a–c) demonstrated I*κ*B phosphorylation and NF-*κ*B nuclear transfer (*n* = 3). (d) Renal pathology of clinical lupus patients with FXR (red) and *α*-SMA (green) double staining (scale bar, 100 *μ*m).

To explore the downstream regulatory mechanisms of low FXR expression in renal tissues in lupus patients, we used the IPA database to retrieve the downstream regulatory targets of FXR (Figure 5(a)), OMIM, TTD, and IPA to search LN-related disease targets. We screened the renal-tissue differential genes of LN patients in GEO database and MRL/lpr mouse differential genes in renal tissues from transcriptome-sequencing studies and then took the overlap of their respective results. The analysis finally focused on the classic inflammation-related NF-*κ*B (Figure 5(b)). We also confirmed by molecular experiments that FXR expression was significantly downregulated and NF-*κ*B transcriptional activity was obviously upregulated in the kidneys of MRL/lpr mice after being reversed by JP intervention (*P* < 0.01; Figures 6(a)–6(c)). Then, we used bioinformatics technology to explore the key targets of JP antagonism against lupus kidney fibrosis and combined the data mining of fibrosis-related disease targets, LN-related microarrays in the public database GEO, and the differential genes of kidney tissue (Figure 5(c)). Finally, we obtained 22 common targets and the expression of these 22 targets in MRL/lpr mouse kidney transcriptome sequencing is shown in the heat map (Figure 5(d)). Combined with relevant literature reports, we found that *α*-SMA may be an important antifibrotic target in JP. We further confirmed through GEO data analysis that *α*-SMA expression was obviously elevated in the kidneys of patients with LN (*P* < 0.01; Figure 5(e)).

### 3.6. JP Can Increase FXR Protein Expression and Inhibit Renal Fibrosis

**Figure 7:**
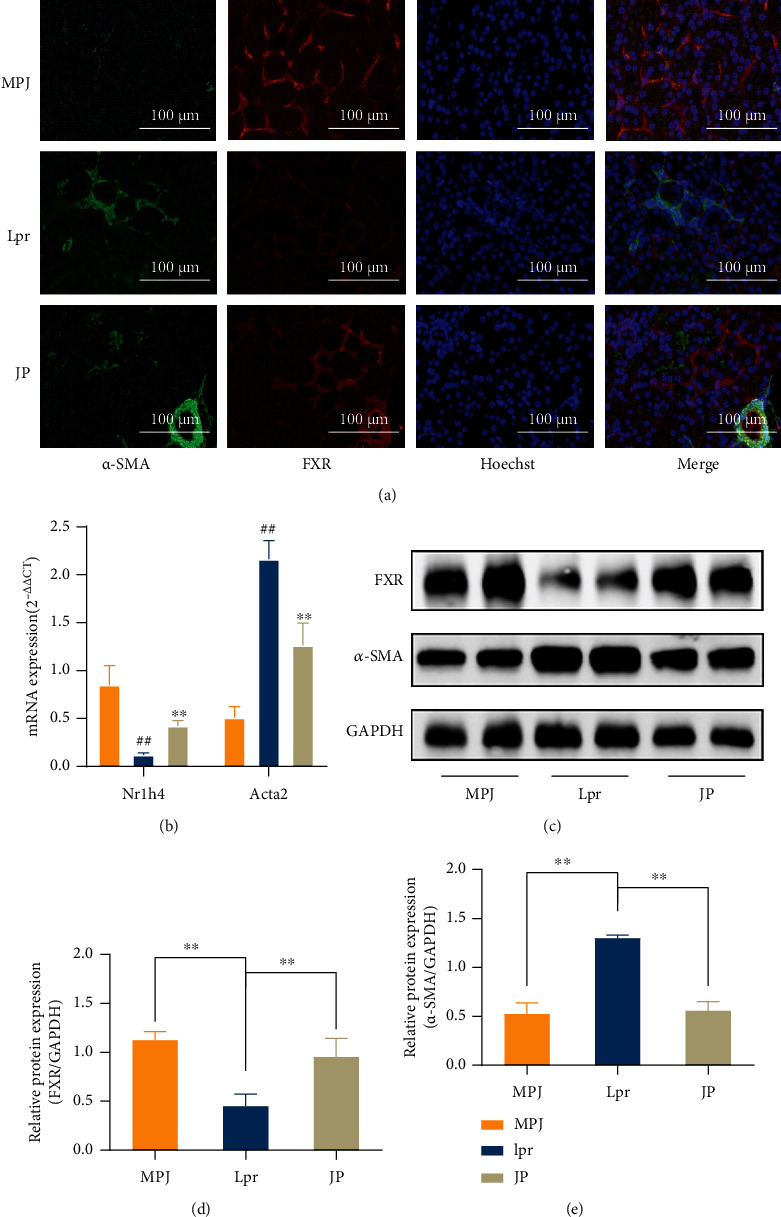
JP can increase FXR expression to suppress *α*-SMA expression at the transcriptional and protein levels. Mouse kidney fluorescence double-staining (a) results demonstrating FXR (red) and *α*-SMA (green) expression (scale bar, 100 *μ*m). Mouse kidney tissue qPCR (*n* = 3) (b) and WB (*n* = 3) (c–e) results were consistent with the fluorescent double-staining results. ^∗∗^*P* < 0.01.

To clarify the relationship between FXR and *α*-SMA in mouse kidneys, we performed immunofluorescence staining of mouse kidney pathological sections. Results showed that FXR and *α*-SMA were expressed around the renal tubules (Figure 7(a)), consistent with the tubular epithelial mesenchymal transition in the renal fibrosis previously studied. FXR significantly decreased, and *α*-SMA significantly increased in the kidneys of mice, but JP reversed this phenomenon. We confirmed this finding by qPCR (*P* < 0.01; Figure 7(b)) and WB technique (*P* < 0.01; Figures 7(c)–7(e)).

### 3.7. FXR Overexpression Reduced the *α*-SMA Protein Content in HK-2 Cells and Was Able to Reduce IL-6 and TNF-*α* Production in LPS-Treated HK-2 Cells

**Figure 8:**
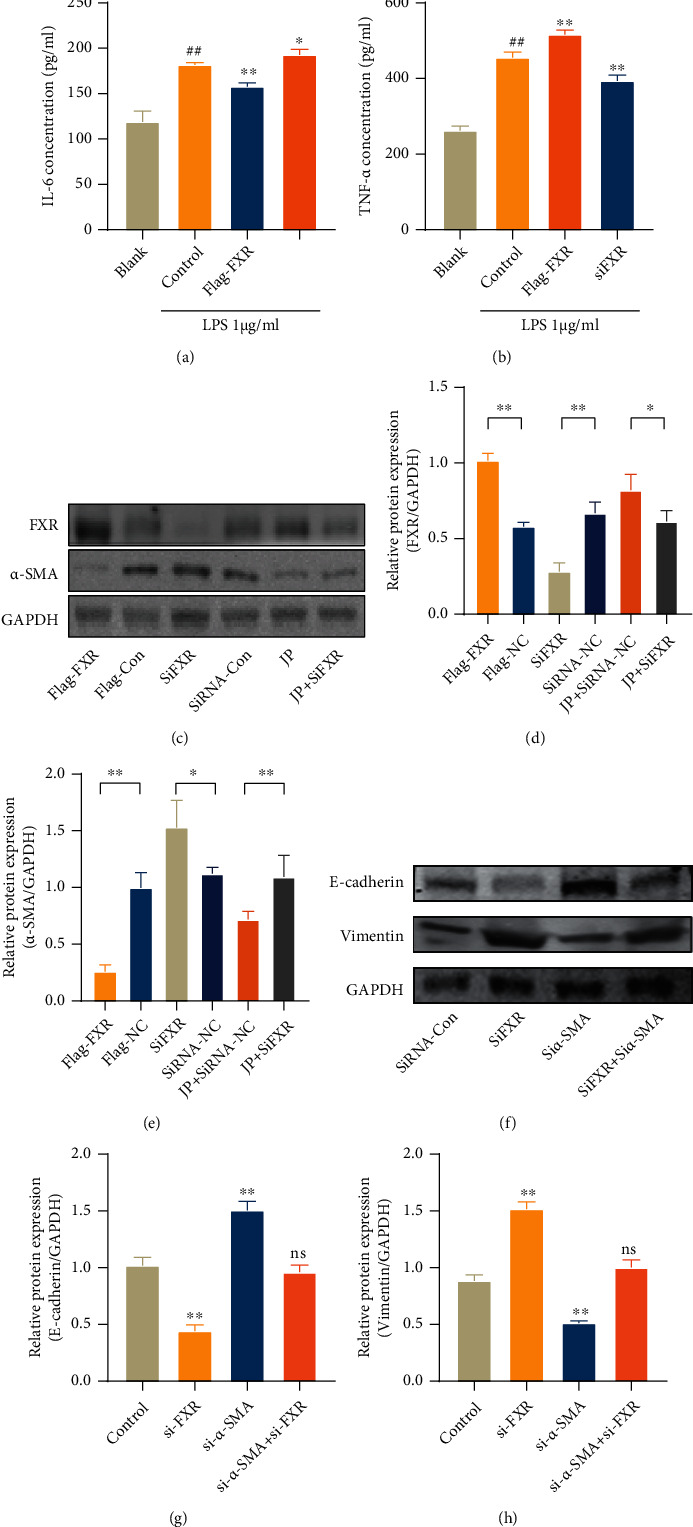
ELISA detected (a, b) the contents of IL-6 and TNF-*α* in the supernatant of HK-2 cells transfected and treated with LPS (1 *μ*g/mL, 6 h). Data are expressed as the mean ± standard deviation (*n* = 3), ^##^*P* < 0.01 versus the blank group; ^∗^*P* < 0.05, ^∗∗^*P* < 0.01 versus the control group. (c–e) WB detection of transfected FXR overexpression or interfering RNA and protein expression of HK-2 cells after stimulation with 10% concentration drug-containing serum and treatment with TGF-*β* (20 ng/mL, 24 h). Data are expressed as the mean ± standard deviation (*n* = 3), ^∗^*P* < 0.05, ^∗∗^*P* < 0.01. WB result (f–h) observation of protein expression after transfection of siFXR or si*α*-SMA and treatment with TGF-*β* (20 ng/mL, 24 h). ^∗∗^*P* < 0.01 versus the control group; ns: not statistically significant.

We used fluorescence staining to observe the FXR and *α*-SMA in kidney tissues of patients with LN (Figure 6(d)), and the results were more consistent with those for MRL/lpr mice; the patient's clinical information is provided in the supplementary materials. We used HK-2 cell to further confirm the regulatory relationship between FXR and *α*-SMA. To further explore the effect of FXR on regulating its downstream NF-*κ*B and alleviating renal inflammation, we observed the effect of FXR on NF-*κ*B transcriptional activity by detecting the IL-6 and TNF-*α* levels. After transfection with FXR overexpression plasmid or siRNA, HK-2 cells were stimulated with 1 *μ*g/mL LPS for 6 h and the released inflammatory cytokines IL-6 and TNF-*α* were observed. Compared with the control group, FXR overexpression can significantly reduce the contents of IL-6 and TNF-*α*, whereas the interference group showed the opposite results (Figures 8(a) and 8(b)). Meanwhile, after FXR overexpression or JP-containing serum treatment, the expression of FXR protein obviously increased and the expression of *α*-SMA protein decreased compared with the control group. However, *α*-SMA expression significantly increased when interfered with FXR (*P* < 0.01; Figures 8(c)–8(e)). These results further confirmed that FXR significantly inhibited *α*-SMA expression, consistent with our previous speculation. We further observed the antifibrotic effect of altering FXR or *α*-SMA protein expression on HK-2 cell by WB experiments. These results showed that E-cadherin expression significantly decreased and vimentin expression significantly increased after transfection of FXR siRNA. Moreover, after *α*-SMA siRNA transfection, EMT was significantly reversed; conversely, cotransfection with FXR siRNA impaired the EMT reversal (Figures 8(f)–8(h)).

## 4. Discussion

SLE is a connective-tissue disease characterized by autoimmunity. It is prevalent among women and is hard to be clinically cured. LN, one of the most serious complications of SLE, can progress to renal failure in advanced stages [12]. Patients with LN have a low life quality and poor prognosis. LN is an important cause of SLE-related death, which places a heavy burden on the patient's family and society [5]. Currently, available therapeutic agents for LN are mostly corticosteroids, antimalarials, and immunosuppressants, which have significant side effects and limited improvement in renal injury despite the improved prognosis for LN patients [13]. JP has been successfully used clinically to treat SLE. Composition analysis confirmed that JP contained a large number of active components with anti-inflammatory, antioxidant, and immunomodulation effects, which may be the material basis for the nephroprotective effect of JP. A previous study has shown that JP can relieve the symptoms related to SLE. It also improves LN and delays the process of kidney tissue fibrosis, but the potential mechanism needs further study.

We used MRL/lpr mice, an internationally recognized classical SLE model with symptoms of autoimmune disease similar to the onset of human SLE [14]. Our results showed that urinary protein, serum anti-dsDNA antibody, and ANA content obviously decreased in MRL/lpr mice after 8 weeks of JP intervention compared with those in model mice. Immune complexes formed by autoantigenic antibodies are also important factors in causing LN [15]. Our study further found that histopathological periglomerular inflammatory cell infiltration was significantly reduced in MRL/lpr mice after JP intervention and glomerular basement-membrane thickening and interstitial fibrosis significantly improved. To explore the mechanism of action of JP in improving LN, we used metabolomics combined with IPA. Serum metabolic differential-enrichment analysis in mice revealed that pathways such as primary bile acid biosynthesis were also altered after treatment with Chinese medicine. Interestingly, a recent study has shown significant changes in serum metabolomics in SLE patients mostly in the metabolic profile. In particular, the conversion of primary to secondary bile acids was affected [16], similar to our findings. IPA further revealed that the JP-group-altered differential metabolite-enrichment core pathway may be closely related to FXR/RXR pathway activation. GEO database microarray results demonstrated that FXR expression was obviously downregulated in lupus patients. Our animal experiments also confirmed that compared with MRL/MPJ mice, the FXR protein content was obviously lower in kidney tissues of MRL/lpr mice, which was obviously reversed by JP intervention.

FXR is recognized as a bile-acid receptor [17]. It is expressed in the liver, kidney, and intestine and participates in glycolipid transport, oxidative stress, and inflammatory processes [18]. Previous research experiments and clinical trials have shown that FXR agonists can be used to treat fatty liver [19] and alleviate liver fibrosis [20–22]. FXR can reportedly mediate multiple pathogenic mechanisms to protect against renal injury in the renal manifestations of various renal diseases and systemic disorders [18, 23]. Studies on human and animal models have shown that FXR predominantly exists in the cortical structure of the kidney [24]. Evidence from several studies has shown that agonists of FXR reduce renal inflammation, oxidative stress, and antirenal fibrosis [18, 25]. For example, they improve diabetic nephropathy [26, 27], promote apoptosis in ischemia-reperfusion kidney injury [28], and inhibit inflammation in acute kidney injury [29]. Interestingly, a preliminary search has revealed rare literature reports of abnormal FXR expression in kidney tissue in the field of LN [30]. For the first time, we confirmed through GEO data analysis and clinical renal-tissue fluorescence staining that FXR expression obviously decreased in the kidney of patients with LN and the degree of inhibition was strongly correlated with disease progression and poor prognosis. These clinical and animal results showed that FXR may play an important nephroprotective role in the progression of LN disease. This finding suggested that JP may play an antagonistic role against LN by mediating FXR regulation in the kidney and the specific mechanism requires further exploration.

**Figure 9:**
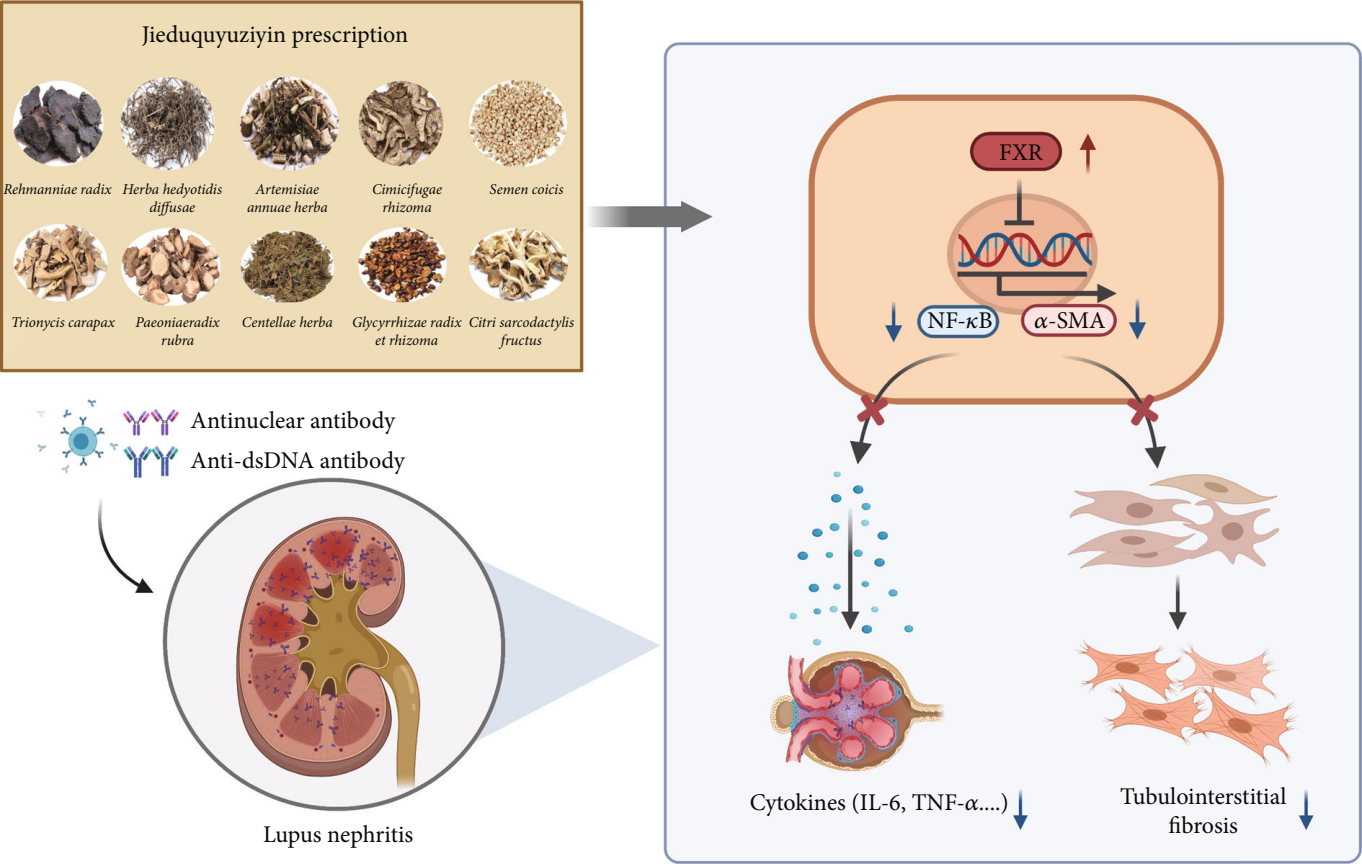
Mechanism diagram. JP decreased renal inflammation and fibrosis by increasing the expression of FXR protein in the kidney, thereby inhibiting its downstream NF-*κ*B and *α*-SMA.

We found that NF-*κ*B and the fibroblast marker protein *α*-SMA may be important downstream targets of FXR regulation through various bioinformatics methods, suggesting that FXR/NF-*κ*B/*α*-SMA-related signaling pathways were perhaps new targets for intervention in LN and renal fibrosis. Our studies have proven that JP can mediate the upregulation of renal FXR expression and inhibit NF-*κ*B and *α*-SMA to exert anti-inflammatory and antifibrotic effects (Figure 9). Many studies have indicated that the process of severe LN leads to end-stage renal injury [31]. The NF-*κ*B family is known to play a necessary role in the progression of LN as key nuclear transcription factors that activate the secretion of inflammatory factors, chemokines [32], etc. The process of NF-*κ*B activation is primarily the degradation of I*κ*B phosphorylation in the cytoplasm and NF-*κ*B entry into the nucleus to activate downstream inflammation-related target-gene expression [33]. Several studies have shown that the use of FXR agonists can inhibit NF-*κ*B protein expression and nuclear translocation [23, 29], consistent with our results showing increased FXR protein expression and decreased I*κ*B protein phosphorylation levels and p65 protein expression in the kidneys of JP-treated mice (Figure 6(a)). The renal biochemical indices and pathological abnormalities of JP-treated mice significantly improved and inflammation levels significantly decreased.

Autoantigens and antibodies in SLE patients combine to form immune complexes deposited in kidney tissue, causing inflammatory reactions that result in kidney cellular dysfunction and eventually to kidney fibrosis [15]. Abnormal and excessive deposition of extracellular matrix (ECM) proteins in the glomerular and interstitial regions is a typical feature of renal fibrosis, and myofibroblasts are the main source of ECM during scar-tissue formation [34]. Myofibroblasts may have multiple sources, such as activated renal fibroblasts, EMT, and fibroblasts [35]. The loss of E-cadherin expression is known to be a marker of EMT, and the upregulated expression of vimentin can be regarded as a marker of cells undergoing EMT. Renal-tissue biopsy studies have shown that *α*-SMA expression is closely associated with LN activity, renal scar formation, and treatment response [15]. In vitro and in vivo experiments manifested that mycophenolate mofetil or rapamycin inhibited various fibrosis markers such as *α*-SMA and improved histopathological abnormalities such as renal tubular EMT, thylakoid proliferation, and progressive glomerulosclerosis [16]. Recent studies have shown that FXR can ameliorate renal fibrosis caused by liver fibrosis [20–22] and unilateral ureteral obstruction models by inhibiting *α*-SMA expression [36]. Some studies have confirmed that FXR activation also inhibits Smad3 expression and regulates the Src-YAP pathway, exerting its antifibrotic effects in multiple ways [14, 26]. We found through ex vivo experiments that JP mediated the upregulation of FXR and that a significant inhibition of *α*-SMA expression could reduce ECM accumulation and slow down the process of LN fibrosis.

## 5. Conclusion

In conclusion, this study showed that JP can likely mediate the activation of renal FXR expression and inhibition of NF-*κ*B and *α*-SMA expression to play a role in suppressing inflammation levels and delaying fibrosis progression for LN prevention and treatment. Our study provided important theoretical insights to develop novel and safe anti-LN drugs for the clinical treatment of SLE by using TCM.

## Data Availability

All data included in this study are available upon request by contact with the corresponding author.

## Abbreviations

SLE: Systemic lupus erythematosus
LN: Lupus nephritis
JP: Jieduquyuziyin prescription
FXR: Farnesoid X receptor
NF-*κ*B: Nuclear transcription factor *κ*B
*α*-SMA: *α*-Smooth muscle actin
UPLC-Q/TOF-MS: Ultraperformance liquid chromatography–quadrupole/time-of-flight mass spectrometry
TCM: Traditional Chinese medicine
ANA: Anti-nuclear antibody
dsDNA: Double-stranded DNA.

## Supplementary Materials

Supplementary Materials We provided clinical information of patients and Western blot images as supplementary materials, the clinical information including various clinical physiological and pathological indicators of patients with lupus nephritis and the control group. And Western blot images contain complete images of Western blot bands.
