# Supplementary material for: Investigating the Mechanisms of Jieduquyuziyin Prescription Improves Lupus Nephritis and Fibrosis via FXR in MRL/lpr Mice
Source: Oxid Med Cell Longev. 2022 Jul 9;2022:4301033. doi: 10.1155/2022/4301033 (PMC9288302; doi:10.1155/2022/4301033)

Western blot images

Figure6C

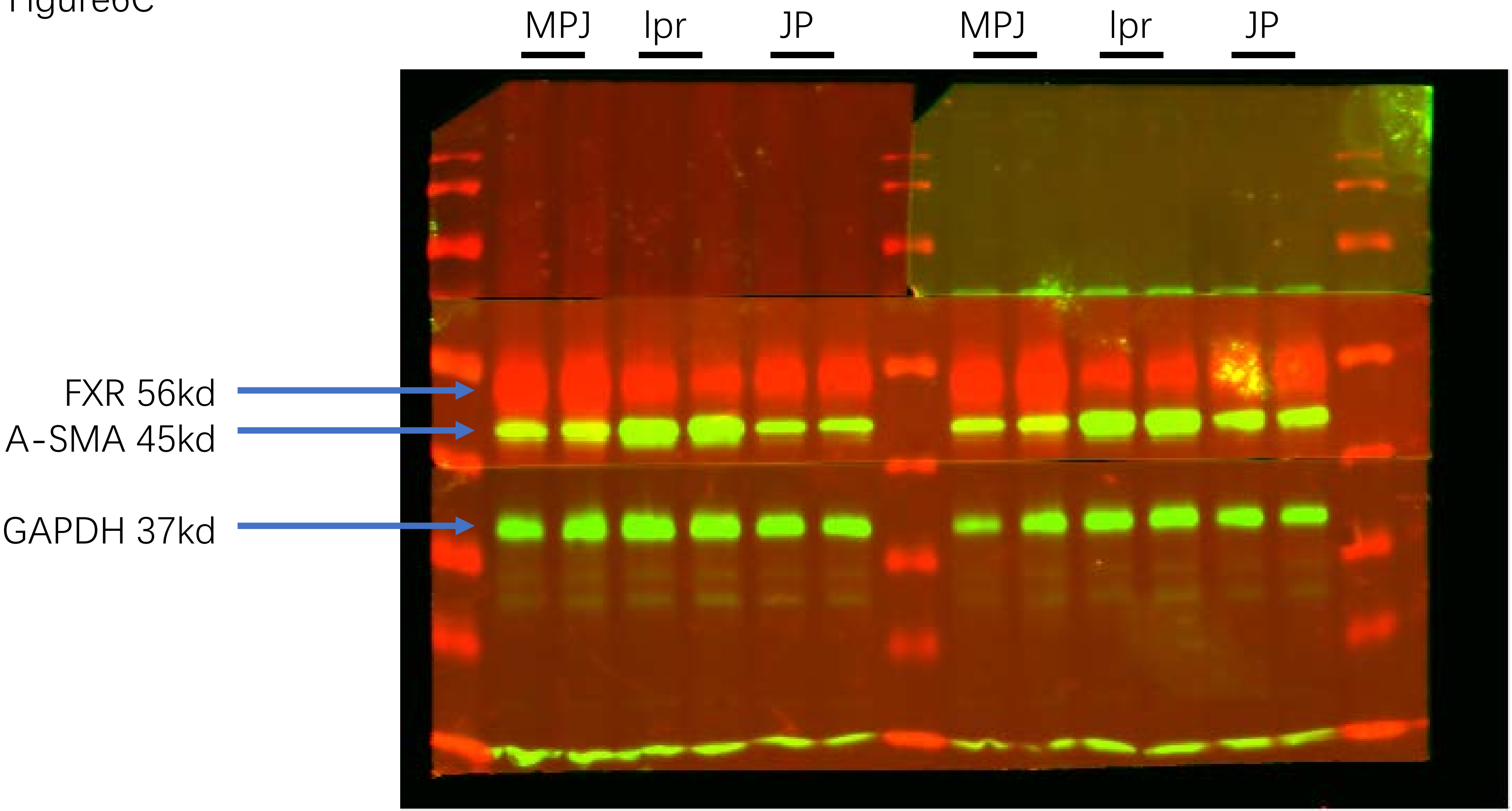

Western blot images

Figure7A

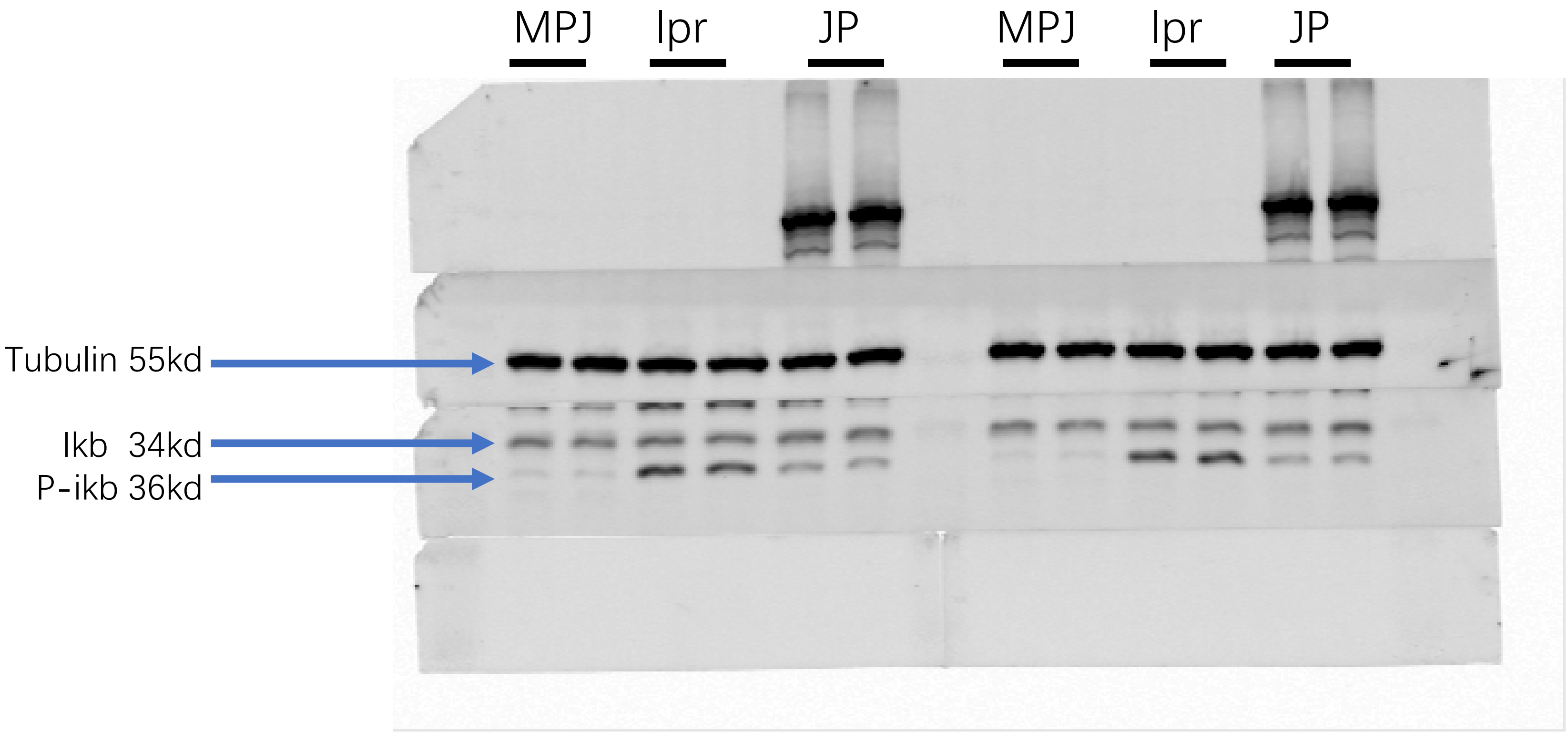

Western blot images

Figure7A

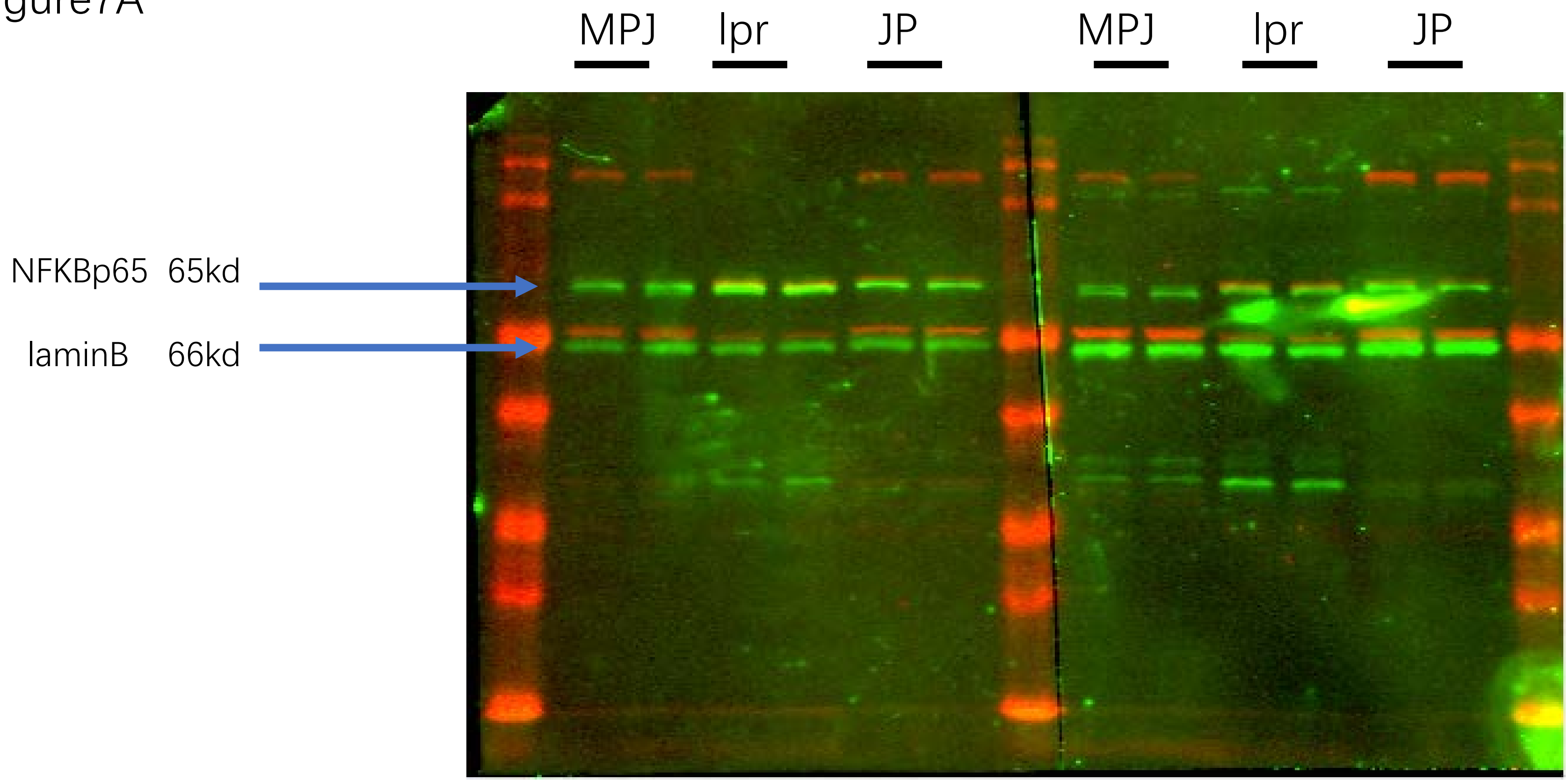

Western blot images

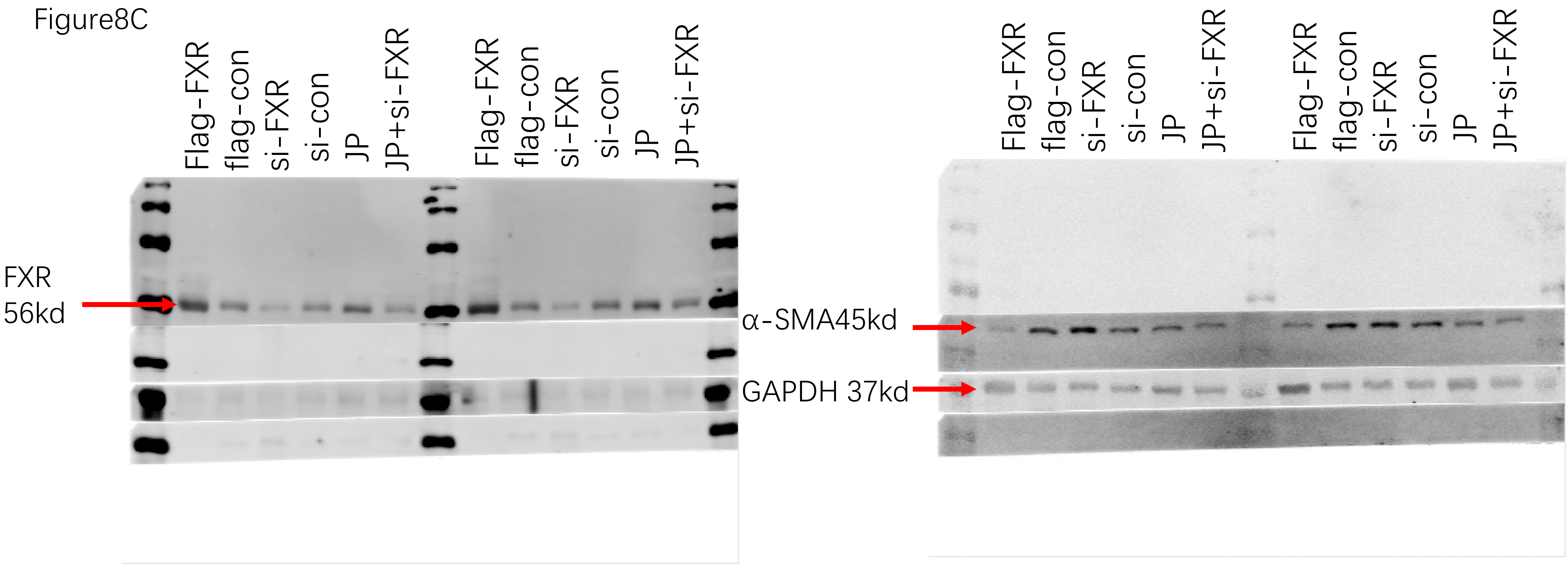

Western blot images

Figure8C

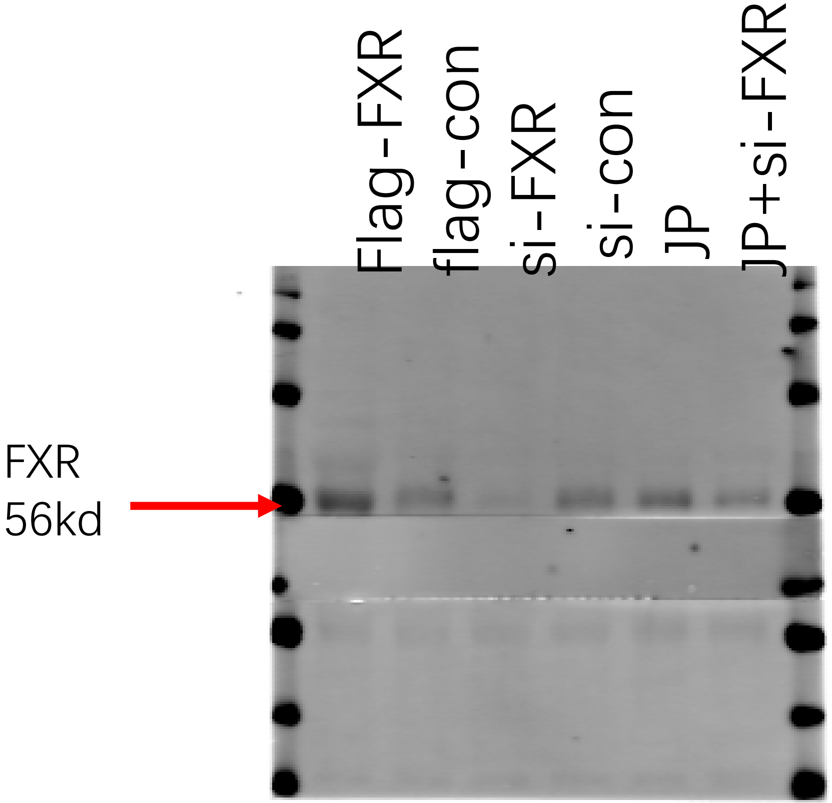

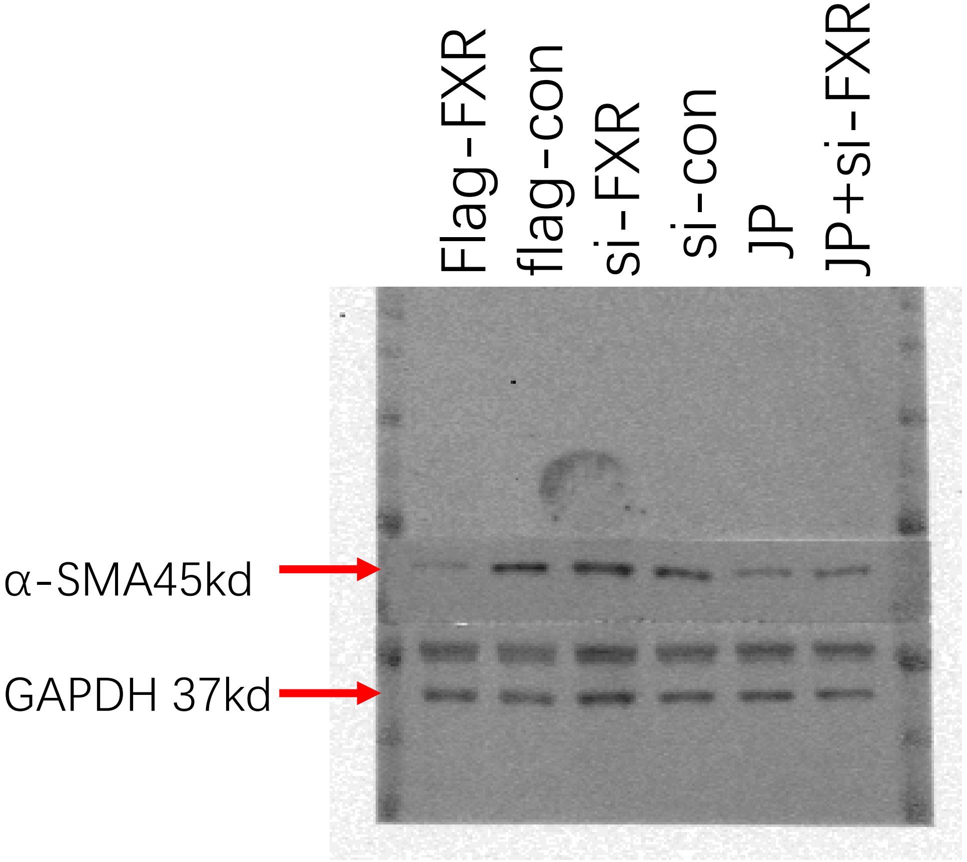

Western blot images

Figure8F

vimentin

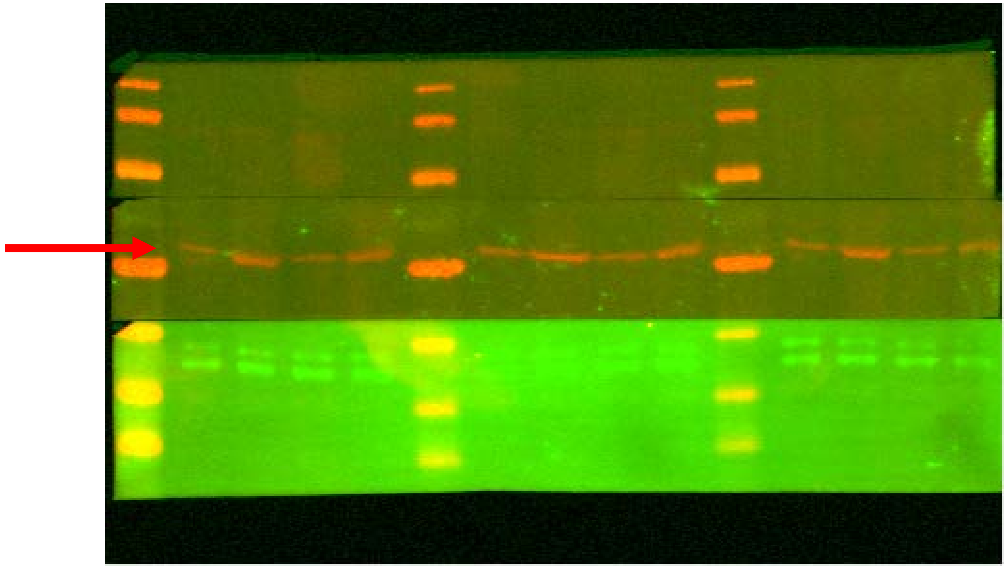

E-cadhderin

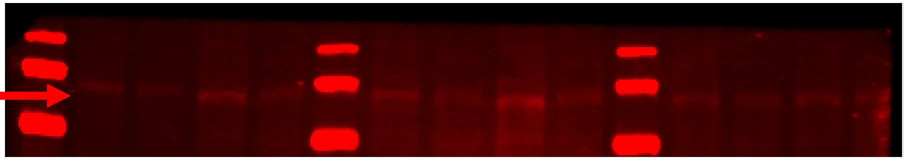

Supplement: Supplementary Materials — We provided clinical information of patients and Western blot images as supplementary materials, the clinical information including various clinical physiological and pathological indicators of patients with lupus nephritis and the control group. And Western blot images contain complete images of Western blot bands. [file 4301033.f1.zip › Western blot images.pdf]
